# Reflections on the first World Field Epidemiology Day

**DOI:** 10.2807/1560-7917.ES.2021.26.36.210909m

**Published:** 2021-09-09

**Authors:** Eurosurveillance editorial team

**Affiliations:** 1 European Centre for Disease Prevention and Control (ECDC), Stockholm, Sweden

Rapid and effective preparedness and response to local outbreaks and pandemics is a mainstay of public health action, and field epidemiologists are key to this endeavour. Field epidemiology training provides fellows with the necessary set of skills to collect, analyse, and interpret data in a consistent and harmonised manner so their results can contribute to evidence-based public health decisions in national and global contexts.

To recognise field epidemiologists and raise awareness about the role they play in the global effort to protect public health, the Training Programs in Epidemiology and Public Health Interventions Network (TEPHINET) organised the first World Field Epidemiology Day (WFED) on 7 September 2021. TEPHINET chose this date because it marks John Snow’s presentation of his findings about the cholera outbreak to local authorities in London on that same day in 1854 [1]. His investigations led to the removal of the handle of a water pump Snow had identified as a possible source of the outbreak, a key event for modern epidemiology and public health.

Along with other national and international organisations, *Eurosurveillance* joined the European Centre for Disease Prevention and Control, the United States’ Centers for Disease Control and Prevention (US CDC) and the Global outbreak alert and response network (GOARN) in signing up as a partner to engage and support WFED. In an editorial published in the International Journal of Infectious Diseases, Martin and Fall summarise 40 years of Field Epidemiology Training Programme initiatives and outline further steps along the Global Field Epidemiology Roadmap to strengthen field epidemiology training worldwide [2].

*Eurosurveillance* has been a platform for timely and authoritative reporting of outbreak investigations over the past 25 years, supporting the work of field epidemiologists and immediate and long-term public health action within Europe and worldwide.

For example, the journal has highlighted and facilitated necessary international collaboration by enabling timely communication surrounding food-borne disease outbreaks. The dedicated anniversary collection, *Eurosurveillance*, 2006–2021: 25 years of public health impact, includes a selection of articles that demonstrate the journal’s impact on food safety as an important area of public health.

In 2018, the ‘disease detective’ work of Swedish and Austrian health authorities, supported by fellows of the European Programme for Public Health Microbiology Training (EUPHEM) and European Programme for Intervention Epidemiology Programme (EPIET), was reported in a rapid communication on a hepatitis A outbreak related to frozen strawberries, highlighting the importance of sequencing in outbreak investigations and increasing awareness of hepatitis A virus infections related to frozen strawberries in Europe [3]. This outbreak was later connected to another when 65 cases across Germany were linked to the same hepatitis A virus strain between 2018 and 2020 [4].

In global contexts, during the Ebola outbreaks in West Africa and the Democratic Republic of the Congo, *Eurosurveillance* published a special edition on Ebola virus disease. This issue shared important public health findings, such as the first secondary case outside of Africa and transmission dynamics and control in Nigeria, and contributed to knowledge transfer and capacity building within and between countries inside and outside of Europe [5,6].

In the interest of evidence-based public health actions to prevent and control infectious diseases and contribute to improved public health, outbreak reporting and sharing experiences from the field will remain a pillar in the future of *Eurosurveillance*.
